# The Evolution of Sex-Related Traits and Genes 2012

**DOI:** 10.1155/2013/590769

**Published:** 2013-03-21

**Authors:** Alberto Civetta, José M. Eirín-López, Rob Kulathinal, Jeremy L. Marshall

**Affiliations:** ^1^Department of Biology, University of Winnipeg, Winnipeg, MB, Canada R3B 2E9; ^2^Department of Cell and Molecular Biology, University of A Coruña, 15001 A Coruña, Spain; ^3^Department of Biology, Temple University, Philadelphia, PA 19122, USA; ^4^Department of Entomology, Kansas State University, Manhattan, KS 66506, USA

The second special issue on the evolution of sex-related traits and genes brings together a wide variety of papers that explore issues dealing with diverse topics such as behavior, organismal defense systems, molecular evolution, speciation, and genomics. This broad diversity reflects the recent expansion of the field of reproductive biology and evolution. The review by R. S. Singh and S. Jagadeeshan provides a historical perspective that describes the breadth of work that has emanated from early protein electrophoresis studies to the genomics-based approaches that are more common today. The authors illustrate how the study of sex- and reproductive-related (SRR) genes has now impacted fields of evolution spanning from selection and speciation to gene birth and evolutionary developmental genetics. 

A review article and a research article address questions related to mating preferences across two very different systems, *Tetrahymena* and *Drosophila*. S. S. Phadke et al. address an interesting question about the evolution of sex: does the evolution of more than one sex necessarily lead to the evolution of mating preferences? Using the ciliate species, *Tetrahymena thermophila*, which has up to seven “sexes,” they tested the mating frequencies of four of the sexes to determine if they exhibited mating preferences. Their results suggest that mating is random among the four sexes, thus concluding that the evolution of multiple sexes does not necessarily lead to the evolution of mate preferences. They discuss their findings in the context of ciliate evolution as well as the evolution of sex in general. A review by A. J. Moehring and M. Laturney offers a clear insight into our current understanding of the genetic basis and evolution of sexual isolation between species. The review focuses on female mate preference as a premating behavioural barrier and highlights two interesting commonalities across a wide variety of *Drosophila* species: different genes control conspecific and heterospecific male choice and a preferential location of genes for heterospecific male rejection in areas of low recombination. Another paper in this issue deals with the question of reproductive isolation between species. J. L. Marshall and N. DiRienzo address a central question in evolutionary biology about whether the same genetic and developmental pathways contribute to reproductive isolation at both intraspecific and interspecific levels. The authors characterize a postmating, prezygotic phenotype, the ability of males to induce egglaying in females, between diverging populations, and species of crickets. Using mating assays and RNAi, the authors demonstrate similar decreases in female fecundity and the abundance of a particular female protein within populations as well as between species. While their results are suggestive of a connection between incompatibilities found within species and reproductive isolation between species, the authors also discuss alternative explanations and the need for future work.

Three papers address a topic not covered in our previous 2011 issue—the relationship between sex and organismal defense systems. In a review article focusing on whether or not different sexes evolve different defensive traits to avoid being harmed or eaten by a predator, G. Avila-Sakar and C. A. Romanow synthesize the literature on the hypothesis of male-biased herbivory in dioecious plant species. They outline shortcomings with the studies supporting the male-biased herbivory hypothesis and suggest a set of alternatives. Moreover, they outline a protocol that they suggest should be used to study plant-herbivore evolution in relation to this important question. Two research articles from L. C. Harshman's lab address questions linked to the evolution of the immune system in *Drosophila*. In one paper, J. Ma et al. compare males and females for measures of metabolic rate and locomotion in populations that have been selected for survival under an infection regime with *Bacillus cereus*. The authors characterize the potential physiological costs associated with mounting an effective and elevated immune response by measuring respiration rates (per fly or adjusted by weight), in addition to behavioural responses as measured by overall fly movement. The authors find evidence of a male-biased response with only the males responding metabolically to selection on elevated immunity. In their other contribution, the authors embark on a selection for survival to infection experiment and identify two pleiotropic responses (i.e., an increase in egg production and delayed development time). Of particular interest to this issue is the identified relationship between reproductive fitness in the form of egglaying and immunity.

Three research articles use molecular biology and evolutionary genomics approaches to study diverse phenomena with respect to sex and evolution. R. L. Kanippayoor and A. J. Moehring address the functional and evolutionary consequences of the timing of protamine expression during the reorganization of the hereditary material in mature spermatozoa. Using transgenic *Drosophila*, they demonstrate that protamines are expressed from both alleles in diploid cells prior to meiosis, in contrast with the postmeiotic haploid expression of mammalian protamines. This work opens the door to future studies to ascertain the evolutionary benefits of diploid *versus* haploid expression of protamines, especially as it pertains to the fertilization success of sperm and an individual's fitness. By characterizing the evolution of the *Izumo* gene family in mammals, P. Grayson and A. Civetta explore how gene duplications can contribute to the adaptations on male reproductive traits. The *Izumo* gene family is comprised of four genes known to be expressed in sperm and possibly involved in sperm-egg interactions. The authors find contrasting patterns of molecular evolution which suggest a variety of evolutionary processes acting across different taxa. They conclude that such differing lineage-specific patterns of selection found in this gene family lend support to protein subfunctionalization as opposed to neofunctionalization or gene loss and represents species-specific adaptations to male fertility. In the final paper of this issue, to identify interlocus conflict in the genome, M. E. B. Hansen and R. J. Kulathinal generate and characterize male- and female-biased networks using the extensive genomic resources available in *Drosophila*. The authors integrate sex-specific expression data from modENCODE with known interaction data to identify putative direct and indirect interactions between male-biased genes and female-biased genes. Using this approach, the authors were able to demonstrate that a larger than expected fraction of the genome may potentially be involved in sexually antagonistic interactions at the molecular level.

This special issue on the evolution of sex-related traits and genes contains a wonderfully diverse sample of studies that addresses both old and new questions in evolutionary reproductive biology. The editors would like to thank the dedicated and generous contributions of all authors and reviewers for helping to make this issue so interesting.

*Alberto Civetta* *Alberto Civetta* *José M. Eirín-López* *José M. Eirín-López* *Rob Kulathinal* *Rob Kulathinal* *Jeremy L. Marshall* *Jeremy L. Marshall*

